# Circular RNA and mRNA profiling reveal competing endogenous RNA networks during avian leukosis virus, subgroup J-induced tumorigenesis in chickens

**DOI:** 10.1371/journal.pone.0204931

**Published:** 2018-10-04

**Authors:** Lingling Qiu, Guobin Chang, Yulin Bi, Xiangping Liu, Guohong Chen

**Affiliations:** 1 College of Animal Science and Technology, Yangzhou University, Yangzhou, PR, China; 2 Poultry Institute, Chinese Academy of Agricultural Sciences, Yangzhou, PR, China; Qingdao Agricultural University, CHINA

## Abstract

Avian leukosis virus subgroup J (ALV-J) can induce myeloid tumors and hemangiomas in chickens and causes severe economic losses with commercial layer chickens and meat-type chickens. Here, we generated ribominus RNA sequencing data from three normal chicken spleen tissues and three ALV-J-infected chicken spleen tissues. Structure analysis of transcripts showed that, compared to mRNAs and lncRNAs, chicken circRNAs shared relatively shorter transcripts and similar GC content. Differentially expression analysis showed 152 differentially expressed circRNAs with 106 circRNAs up regulated and 46 circRNAs down regulated. Through comparing differentially expressed circRNA host genes and mRNAs and performed ceRNA network analysis, we found several tumor or immune-related genes, in which, there were four genes existed in both differentially expressed mRNAs and circRNA host genes (*Dock4*, *Fmr1*, *Zfhx3*, *Ralb*) and two genes (*Mll*, *Aoc3*) involved in ceRNA network. We further characterized one exon-intron circRNA derived from *HRH4* gene in the ceRNA network, termed circHRH4, which is an abundant and stable circRNA expressed in various tissues and cells in chicken and localizes in cytoplasm. Our results provide new insight into the pathology of ALV-J infection and circRNAs may also mediate tumorigenesis in chicken.

## Introduction

Avian leukosis virus (ALV) is an avian oncogenic retrovirus, which belongs to genus *Alpharetrovirus*, subfamily *Orthoretrovirinae* within the *Retroviridae* family, leads to neoplastic diseases and other reproduction problems in the poultry industry worldwide. It can be classified as endogenous virus (subgroup E) or exogenous virus (subgroup A, B, C, D, and J) according to viral envelope interference, host range, cross-neutralization patterns and mode of transmission[1]. ALV-J virus was first isolated in 1988, reported in 1991[2] and broke out in meat-type and egg-type chickens during 2000s [3–5]. ALV-J infected hosts are characterized as delayed growth, immune tolerance, high mortality, a diversity of tumors, enhanced susceptibility to secondary infection, which result in enormous financial losses in poultry industry worldwide since 1990s [6–8].In China, ALV-J infection has become epidemic and induced severe outbreaks in both commercial layer chickens and meat-type chickens [9–11]. The morbidity and mortality rates caused by ALV-J infection have reached 60% and 20%, respectively [12]. The more effective way to deal with ALV-J infection now is to control and eradicate it from breeding chicken farm, which costs too much [13].

Circular RNAs (circRNAs) are a naturally occurring family of noncoding RNAs that is highly represented in the eukaryotic transcriptome [14, 15]. circRNAs had generally been considered as errors in splicing without biological functions, but along with high-throughput sequencing and precise computational approaches developing, emerging evidence suggests that they are widespread and substantial presence within transcriptome [14, 16, 17] and their function as key regulators in plentiful biological processes, for example, neural development, cell growth, as well as different types of cancer [18–21]. Recently, circRNAs have been shown to act as microRNA (miRNA) sponges to regulate gene expression and function in many biological processes [16, 22]. Sometimes, one circRNA could harbor multiple miRNA-binding sites and inhibit miRNA activity to bind mRNA targets to serve as competing endogenous RNAs (ceRNAs) [21, 23]. However, only a few circRNAs contain multiple binding sites to trap one particular miRNA [24], and the function of circRNA remains largely unknown.

In chicken, circRNAs have been charachterized in myoblast and liver tissue [25, 26]. In this study, we generated ribominus RNA sequencing data from three normal chicken spleen tissues and three ALV-J-infected chicken spleen tissues, and identified 4254 circRNA candidates, in which, 152 circRNAs were differentially expressed between two groups with 106 circRNAs up regulated and 46 circRNAs down regulated. Analysis of these circRNAs revealed that one gene could produce multiple circRNAs in chicken and chicken circRNAs, like lncRNAs, shared relatively shorter transcripts and similar GC content to protein-coding transcripts. Differential expression analysis and ceRNA network analysis showed several tumor-associated transcripts (Dock4, Fmr1, Zfhx3, Ralb, Mll, Aoc3, and circHRH4) may involve in ALV-J-induced tumorigenesis. We further characterize one abundant circRNA produced from the *HRH4* gene, termed circHRH4. Our findings indicate that circRNAs may also mediate ALV-J-induced tumorigenesis in chicken.

## Materials and methods

### Ethics statement

All experimental procedures were performed in accordance with the Regulations on The Administration of Experimental Animals issued by the Ministry of Science and Technology in 1988 (last modified in 2001, Beijing, China). All experimental animal operations were approved and guided by the Animal Care and Use Committee of Yangzhou University.

### Sampling

Twenty-week-old female black-bone silky fowls (BSFs) with or without spontaneous ALV-J infection, but without contamination of other tumorigenic viruses in avian: Marek's disease virus (MDV) [27] and reticuloendotheliosis virus (REV) [28] detected by polymerase chain reaction (PCR), were obtained from the progenitor breeding chicken farm of Lihua Animal Husbandry Co. Ltd. (Jiangsu, China). The total RNAs of two groups spleen tissues (ALV-J-positive group: ALV-J+; ALV-J-negative group (uninfected goup): ALV-J–) were obtained and subjected to RNA sequencing (RNA-Seq) and small RNA-Seq. In the meantime, we also chose additional four normal and four ALV-J-infected chickens after pathological and genetic review and then collected normal spleen tissues and ALV-J-infected spleen tissues for key genes and circRNAs validation and other six normal tissues (heart, liver, lung, lymph gland, bursa fabricius and kidney) were obtained to analyze tissue expressional profile.

### RNA-seq analysis

All RNA samples were first treated to remove genomic DNA and rRNA, and then construct library and finally, be sequenced by HiSeq 2500 (Illumina, San Diego, California). Sequencing data were submitted to the NCBI GEO database with the accession number GSE118752.

For small RNA-Seq, small RNA fractions were ligated to 5′ and 3′ RNA adaptors. Subsequently, cDNA libraries (150 bp) were constructed and then underwent quantification and quality assessment by Qubit 2.0 fluorometer and Agilent 2100 Bioanalyzer (Agilent). Finally, the libraries were sequenced by HiSeq 2500 (Illumina). Sequencing data were submitted to the NCBI SRA database with the accession number SRP158563.

### Identification and quantification of chicken circRNAs

FASTQ reads were mapped to the chicken reference genome (Gallus gallus 4.0, April 2013, Ensembl Build 85) with TopHat (version: 2.0.9) [29]. The transcripts were assembled with Cufflinks program [30] (v2.2.1). The fragments per kilobase of transcript per million mapped reads (FPKM) (Trapnell et al., 2010) was used to estimate each gene expression.

All unmapped reads were then used to identify circRNAs using CIRI algorithm [31]. Briefly, the CIRI detected junction reads with paired-end mapping (PEM) and GT-AG splicing signals. The total number of reads with back-spliced junctions was used to measure circRNA abundance, and FPKM was used to estimate relative expression of a circRNA.

### Bioinformatics analysis

Differentially expressed circRNAs were identified between different groups with the R package DESeq with a *P*-value < 0.05. Gene Ontology (GO) and Kyoto Encyclopedia of Genes and Genomes (KEGG) functional enrichment analyses were performed with the GOseq package, and terms and pathways with corrected *P*-value of less than 0.05 were considered as being significantly enriched. Differentially expression and enrichment analyses of mRNAs and miRNAs were also performed in our previous study as described above [32].

For ceRNA analysis, potential miRNA response elements (MREs) were searched in circRNA and mRNA sequences with miRanda software, and circRNAs and mRNAs shared the same MREs and relatively high MRE density were considered to predict circRNA–miRNA–mRNA interactions [33]. miRNA-circRNA/mRNA co-expression was identified when PCC ≥ 0.7 or ≤ –0.7 and p-value ≤ 0.05.

### Cell culture and treatments

The chicken HD11 macrophage cell line was provided by Prof. Susan J. Lamont, Iowa State University, USA. DF-1 cells were obtained from American Type Culture Collection (ATCC). HD11 cells were cultured in RPMI1640 medium (Hyclone, USA) supplemented with 10% fetal bovine serum (FBS) (Gibco, USA), 5% chicken serum (Gibco), 10 mM HEPES—(Gibco), 2 mM L-glutamine (Gibco), 0.1 mM non-essential amino acids (Gibco), 1 mM sodium pyruvate (Gibco) and 0.05 mM β-mercaptoethanol (Sigma Aldrich, USA) and DF-1 cells were cultured in Dulbecco’s modified Eagle’s medium supplemented with 10% FBS at 37°C with an atmosphere of 5% CO_2_ and 60–70% relative humidity.

Transcription was blocked by adding 2 μg/ml actinomycin D or dimethylsulphoxide (Sigma-Aldrich, St Louis, MO) as a control to the cell culture medium and cells were harvested at 0, 4, 8, 12 and 24 h and then analyzed the cell cycle progression and apoptosis with Cell Cycle and Apoptosis Analysis Kit (Beyotime, Jiangsu, China). Briefly, harvested cells were fixed overnight with 70% ethanol and incubated with RNase for 5 min, and then incubating with propidium iodide (PI) in the dark for 30 min at 37 °C. The DNA content was analyzed using a FACS Calibur flow cytometer (Becton Dickinson, Oxford, UK). RNA preparation.

The nuclear and cytoplasmic fractions were extracted using NE-PER Nuclear and Cytoplasmic Extraction Reagents (Thermo Scientific). Total RNA from whole-cell lysates or tissues or the nuclear and cytoplasmic fractions were isolated using TRIzol (Life Technologies, Carlsbad, CA). For RNase R treatment, 2.5 μg of total RNA was incubated with 3 U/μg of RNase R (Epicentre Technologies, Madison, WI) or RNase-free water as a control 30 min at 37 °C and then extinguishing enzyme activity 10 min at 70 °C.

### qRT-time PCR analysis

qRT-PCR analysis was conducted using SYBR Premix Ex Taq II (TaKaRa, Shiga, Japan) and QuantStudio 5 real-time PCR instrument after cDNA synthesis with the PrimeScript RT Reagent Kit with gDNA Eraser (TaKaRa). Primer sets were designed with Primer-BLAST (http://www.ncbi.nlm.nih.gov/tools/primer-blast/), using differentially expressed transcript sequences found by sequence analysis, and synthesized by Tsingke Biotech (Beijing, China). Two reference genes (GAPDH and SDHA) were detected as internal controls. All assays were run in triplicate (primer sequences are provided in Table 1).

**Table 1:** Sequences of primers used to amplify common avian viruses.

Primer name	Primer sequence (5’-3’)	Length/bp
Dock4	F: CCTGACAAAGCTGTAAACGCC	256
	R: CCGGGGAGTTGGATATGAGC	
Fmr1	F: TCTACTGATCAGAATTGACTGCAA	146
	R: GCTGTTGACCATCTGCGTTG	
Zfhx3	F: AGACTAGATCCCGAACTGCTG	170
	R: TGCGTTTCGTGTTCTCACTTC	
Ralb	F: CGGGTCGTAGTCTTTAGGTCT	159
	R: CTGGAGAGTGAGCGCAGATT	
Mll	F: GCAGTGGACATGTGGAGTTTG	87
	R: CACTTGTTACACTCCAGCAGC	
Aoc3	F: CGGGAAGGTGCCGTACCA	187
	R: GCCTTCCTCAGCTCCTTCTG	
circ2573	F: AGTTCTGCCTGTGGCGATAC	159
	R: TGAGGTTTGGGGTGATCTGG	
circ1193	F: CTCGGAAACGTCCTGGTGAT	173
	R: CATAGCCCTTTCCCCAGGTG	
circ0491	F: TGGGTCAGCCATTGAGAACTT	269
	R: TGTCGTAGAACTGGATCAGGGA	
Hrh4	F: GCCTTCCTGCTCTATTCTCCA	296
	R: CCCTCAGCACCAAGAACGG	
GAPDH	F: CGATCTGAACTACATGGTTTAC	151
	R: TCTGCCCATTTGATGTTGC	
SDHA	F: CAGGGATGTAGTGTCTCGT	187
	R: GGGAATAGGCTCCTTAGTG	

### Statistics

Statistical analyses of qRT-PCR data were performed using GraphPad Prism 6 software (GraphPad Software Inc., San Diego, CA) and presented as the mean±SEM. *P* < 0.05 was considered to represent a statistically significant difference.

## Results

### Profiling of circRNAs in chicken normal and ALV-J-infected tissues

**Fig 1:**
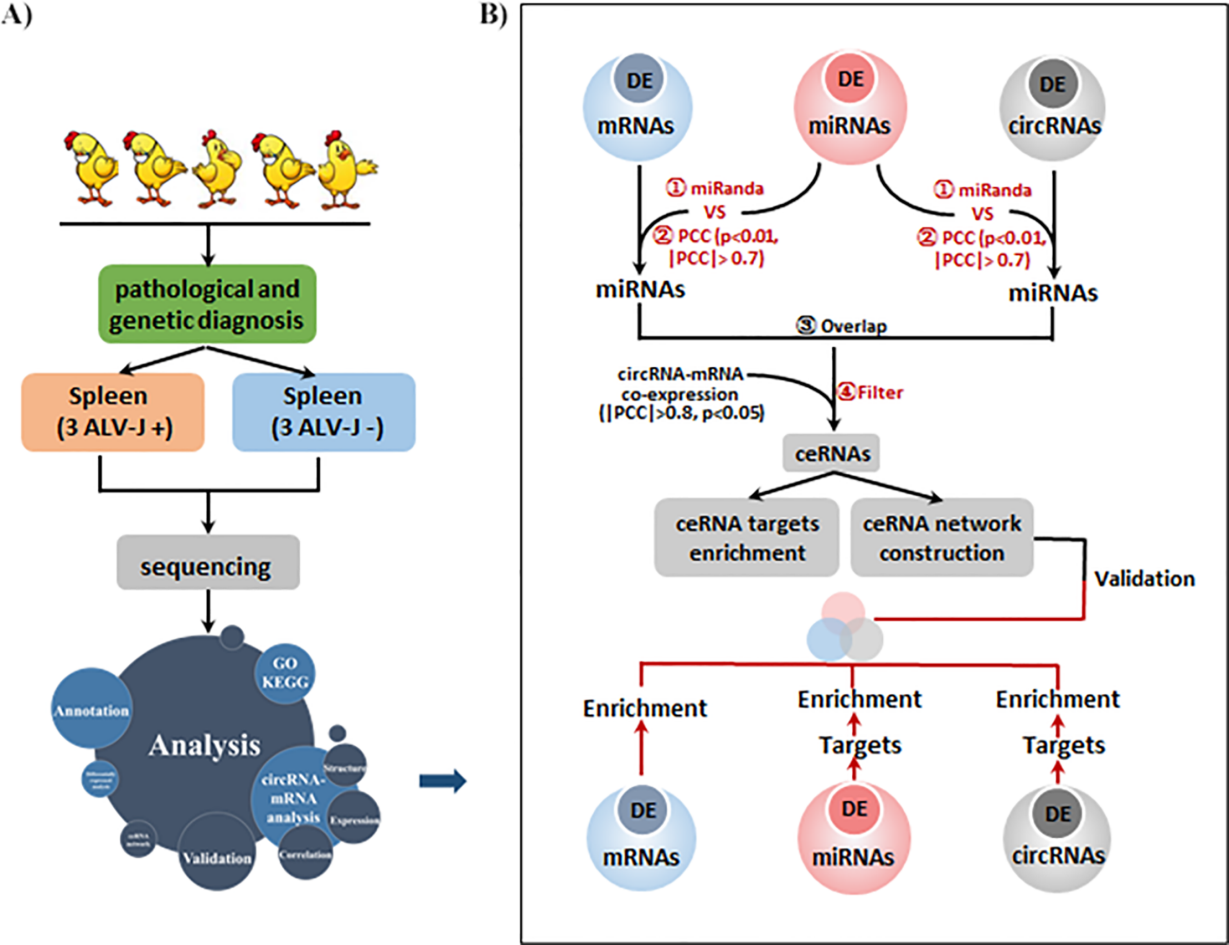
A schematic representation of the study design and computational analysis.

First, we characterized circRNA transcripts using RNA-seq analyses of ribosomal RNA-depleted total RNA from three normal chicken spleen tissues and three ALV-J-infected chicken spleen tissues. Each sample was sequenced on an Illumina HiSeq 2500, then yielded reads were mapped to the chicken reference genome (Gallus gallus 4.0, April 2013, Ensembl Build 85) by TopHat (version: 2.0.9). A schematic representation of the study design and computational analysis was shown in Fig 1.

**Fig 2:**
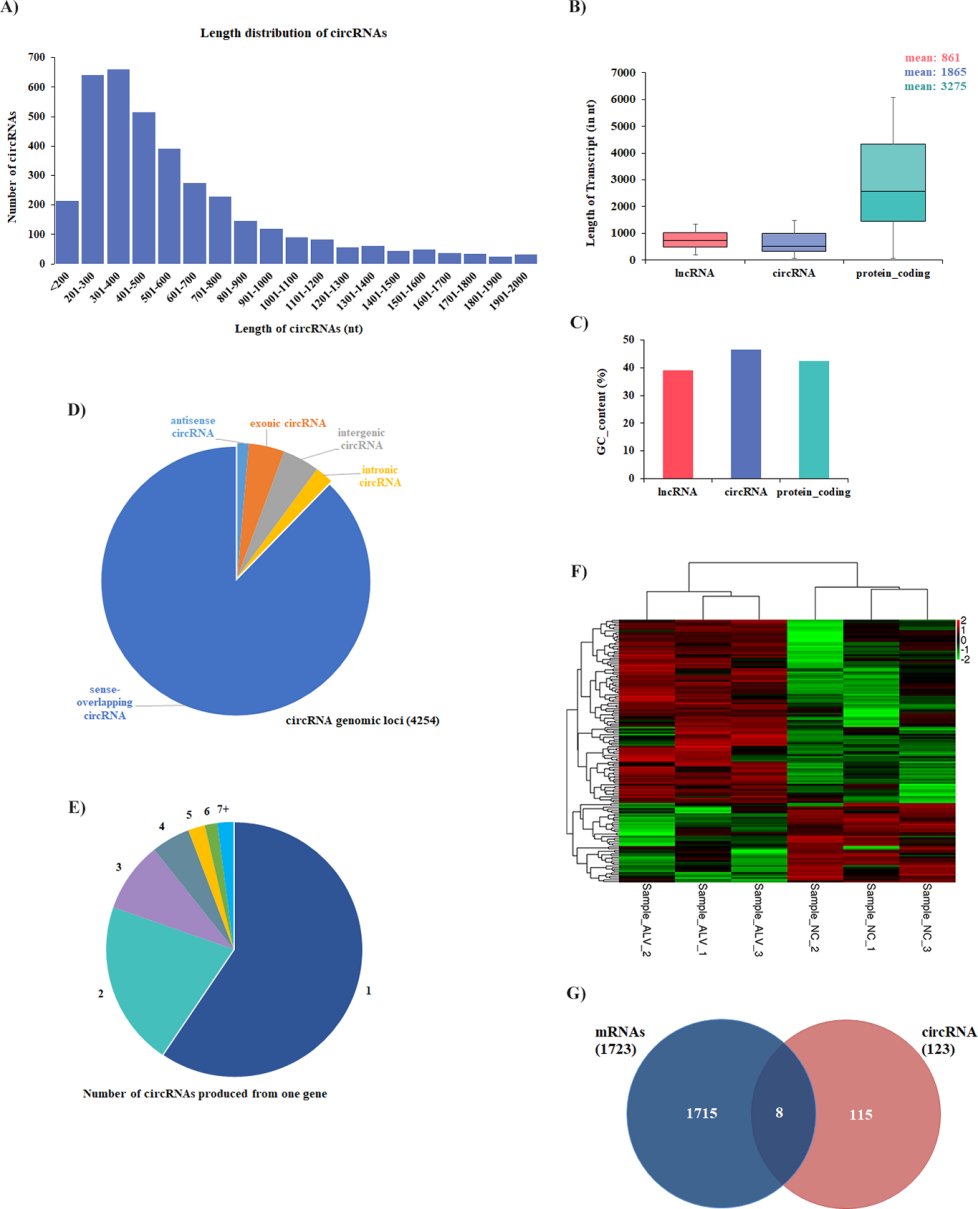
Profiling of circRNAs in chicken normal and ALV-J-infected tissues. **(A)** Length distribution of circRNAs. **(B)** Transcript length comparison, chicken circRNAs was about one-second of the length of protein-coding transcripts, and twice the length of lncRNAs (mean length of 861 nt for lncRNAs; 1865 nt for circRNAs; 3275 nt for protein-coding transcripts). **(C)** GC content, lncRNA shows in red, circRNA in purple and protein-coding transcripts in green, the average GC content of three kinds of transcripts were almost equal (mean GC content of 46% for circRNAs; 40% for lncRNAs; 42% for protein-coding transcripts). **(D)** Genomic origin of chicken circRNAs. **(E)** Number of circRNAs produced form one gene in chicken (4,254 circRNAs from 2,191 host genes). **(F)** Heatmaps of 152 significantly differentially expressed circRNAs show normalized expression values. Columns represent samples, and rows represent transcripts. Colors are used to represent expression levels: above (red) or below (green) (expression data was normalized from -2 to +2). Sample_ALV: ALV-J infected tissues; Sample_NC: normal uninfected tissues. **(G)** Venn diagrams of differentially expressed mRNAs, circRNA host genes.

In total, 4254 circRNA candidates were identified in chicken spleen tissues. Transcript-length analysis showed that the length of most circRNAs was less than 500 nucleotide (nt) and the median length was ~400 nt (Fig 2A), while the length of most human circRNAs was less than 1500 nt and median length was approximately 500 nt. Comparing the length of different transcripts (lncRNAs, circRNAs, and protein-coding transcripts), we found that the mean length of chicken circRNAs was about one-second that of protein-coding transcripts, and twice length of lncRNAs (mean length of 861 nt for lncRNAs; 1865 nt for circRNAs; 3275 nt for protein-coding transcripts) (Fig 2B). In addition, the average GC content of circRNAs (approximately 46%) was almost equal to that of lncRNAs (approximately 40%) and protein-coding transcripts (approximately 42%) (Fig 2C). Structure analysis of circRNAs showed that more than 80% of chicken circRNAs were sense-overlapping, whereas smaller fractions aligned with introns, exons, and antisense regions to known transcripts (Fig 2D). Analysis of the number of circRNAs from their host genes revealed that one gene could produce multiple circRNAs in chicken (Fig 2E, 4,254 circRNAs from 2,191 host genes).

Then we compared the expression levels of circRNAs between the ALV-J-infected group and the uninfected group, and identified 152 circRNAs that were significantly differentially expressed between ALV-J-infected and uninfected chickens (*P*-value < 0.05). Of these circRNAs, 106 (approximately 69.7%) were upregulated and 46 (approximately 30.3%) were downregulated. We further created a heat map of differentially expressed circRNAs (Fig 2F). Comparison of independently clustered expression profiles of circRNAs revealed that differentially expressed circRNAs could be grouped into two broad classes: (I) circRNAs (approximately 30%) that were present in normal uninfected tissues and decayed in ALV-J-infected tissues; and (II) circRNAs (approximately 70%) that were absent or present at low levels in normal uninfected tissues and were induced at high expression levels in ALV-J-infected tissues.

By comparing differentially expressed mRNAs, and host gene of circRNAs, we found that they shared eight common genes (Fig 2G), including Dedicator of cytokinesis 4 (*Dock4*); Fragile X Mental Retardation 1 (*Fmr1*); Zinc Finger Homeobox 3 (Zfhx3) RAS like proto-oncogene B (*Ralb*). The DOCK4 functions as a guanine nucleotide exchange factor and is involved in regulation of adherens junctions between cells and in cell migration, could suppresses tumor invasion in osteosarcoma mouse cell lines [34–36]. FMR1 may be involved in the regulation of ATR-dependent signaling pathways such as BRCA1 phosphorylations and interact with BRCA in human Ovarian Cancers [37]. ZFHX3 may function as transcriptional regulator and was reported to participate in gastric cancer by regulating MUC5AC promoter [38]. RALB was reported to involve in various cellular processes including cell migration, proliferation, suppression of apoptosis, and oncogenic transformation [39, 40].

### GO and KEGG enrichment analyses of the host genes of the differentially expressed circRNAs

**Fig 3:**
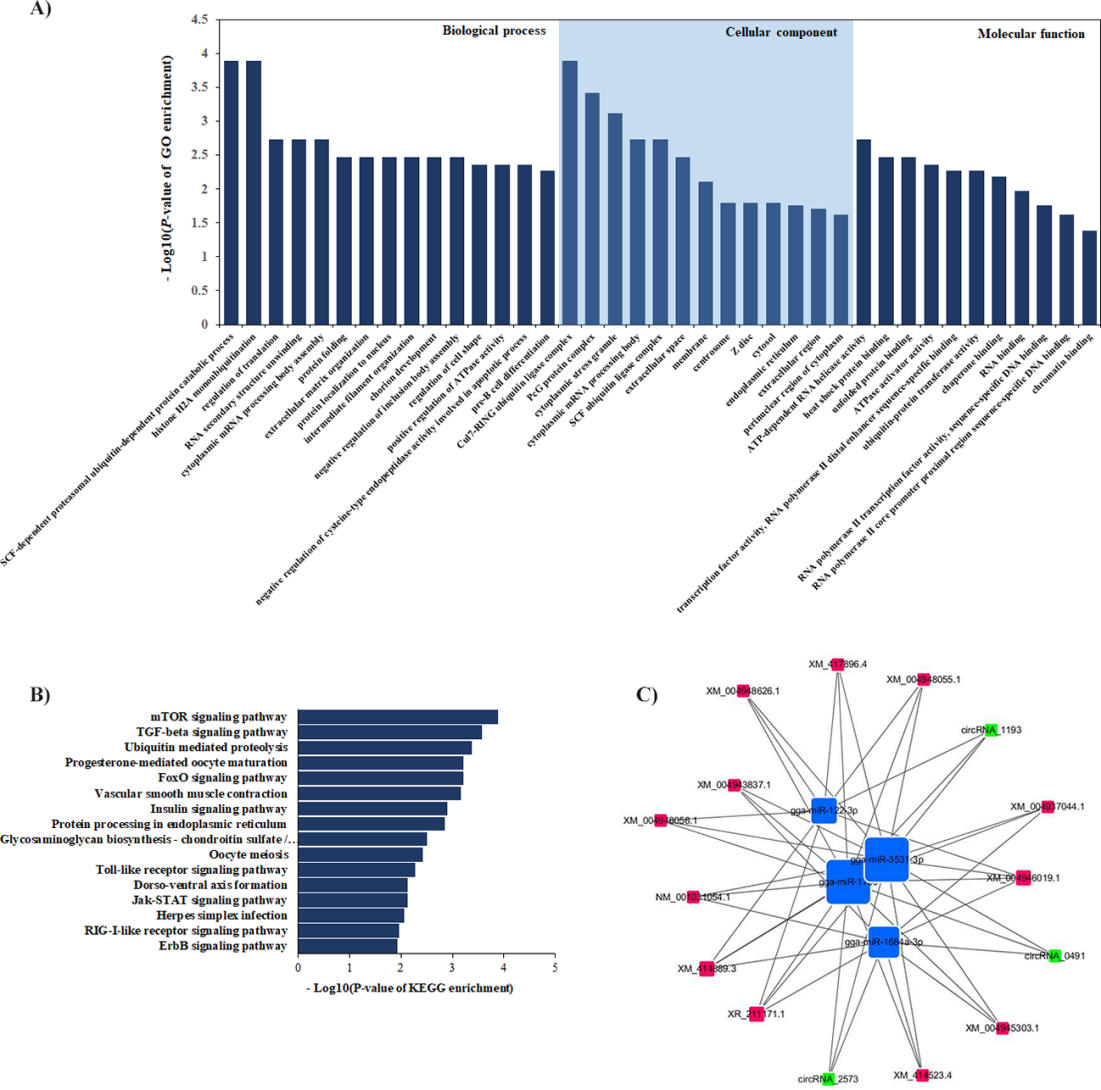
Functional annotation of differentially expressed circRNAs between ALV-J-infected and uninfected samples. **(A)** GO analysis of differentially expressed circRNAs. **(B)** KEGG pathway analysis of differentially expressed circRNAs. **(C)** ceRNA networks between differentially expressed circRNAs and mRNAs.

Functional annotation was performed by GO and KEGG pathway analyses to determine the biological significance of host genes of differentially expressed circRNAs in ALV-J-infected chickens. GO enrichment analysis of differentially expressed circRNAs involved in pre-B cell differentiation, negative regulation of cysteine-type endopeptidase activity involved in apoptotic process, and several protein processing-related terms (Fig 3A). KEGG pathway enrichment analysis of significantly dysregulated circRNAs showed significant enrichments in cell-growth, immune and cancer-associated pathways, such as the mTOR signaling pathway, TGF-beta signaling pathway, Toll-like receptor signaling pathway, RIG-I-like receptor signaling patheway, Jak-STAT signaling pathway, Insulin signaling pathway and the ErbB signaling pathway (Fig 3B). The details of these GO terms and pathways were provided in S1 and S2 Tables, respectively.

### Construction of ceRNA networks

Through ceRNA analysis, we constructed mRNA–lncRNA crosstalk networks Based on the ceRNA network, we found 92 circRNA–miRNA–mRNA co-expression links (Fig 3C), which including three circRNAs serving cooperatively as ceRNAs and four miRNAs targeting twelve genes. Network-related genes including several tumor-associated and immune-related genes, such as myeloid/lymphoid or mixed-lineage leukemia (*Mll*); Amine Oxidase, Copper Containing 3 (*Aoc3*); MLL, also known as KMT2A, was reported to inhibit GADD34-induced apoptosis [41]and chromosomal aberrations involving MLL are a cause of certain acute lymphoid leukemias and acute myeloid leukemias [42, 43]. AOC3 (also known as VAP-1) was reported as a cell adhesion protein that participated in lymphocyte extravasation and recirculation [44]. These genes and their related miRNAs/circRNAs links got our attention, which could play roles in ALV-J-induced tumorigenesis in chicken.

### Verification of differentially expressed transcripts by qRT-PCR

**Fig 4:**
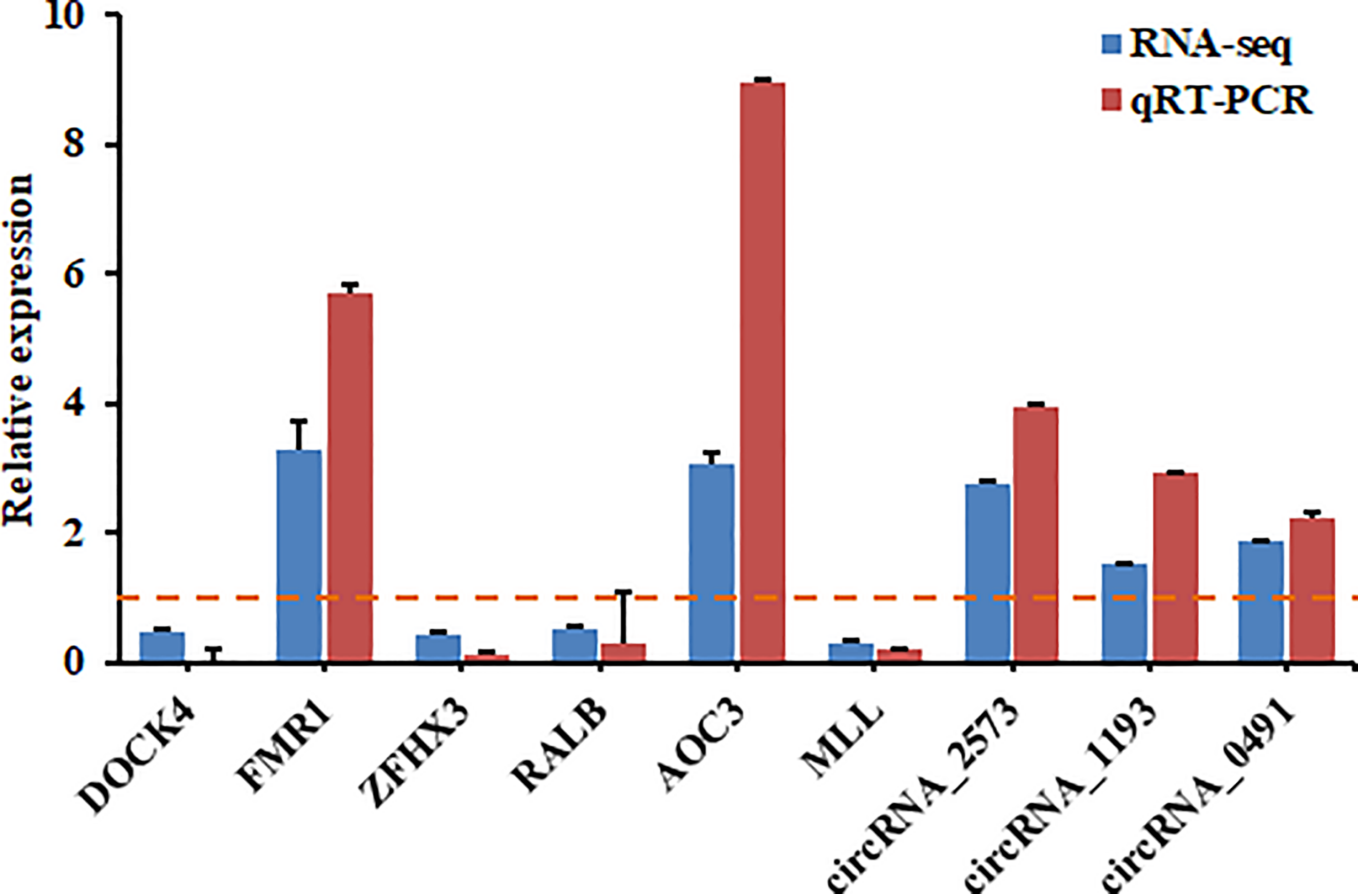
qRT-PCR confirmation candidate transcripts and comparison with sequencing results in chickens with or without ALV-J infection.

To validate the expression of these differentially expressed transcripts if they are proposed as candidate transcripts for future studies on the pathology of ALV-J infection in chicken, we obtained independent chickens to collect spleen tissues from four adult normal and four ALV-J-infected chickens and detected expression levels of seven genes (*Dock4*, *Fmr1*, *Zfhx3*, *Ralb*, *Mll*, *Aoc3* and *Hrh4*) and three circRNAs (circRNA_2573, circRNA_1193, and circRNA_0491) in extra normal and ALV-J-infected spleens by qRT-PCR and compared the results with sequencing results (Fig 4). The uninfected group was used as control group, and the infected group with values < 1 indicating downregulated expression and values > 1 indicating upregulated expression. The results showed that both two methods showed consistent results in terms of the transcripts’ upregulation or downregulation.

### The characteristics of circHRH4 RNA in chicken

**Fig 5:**
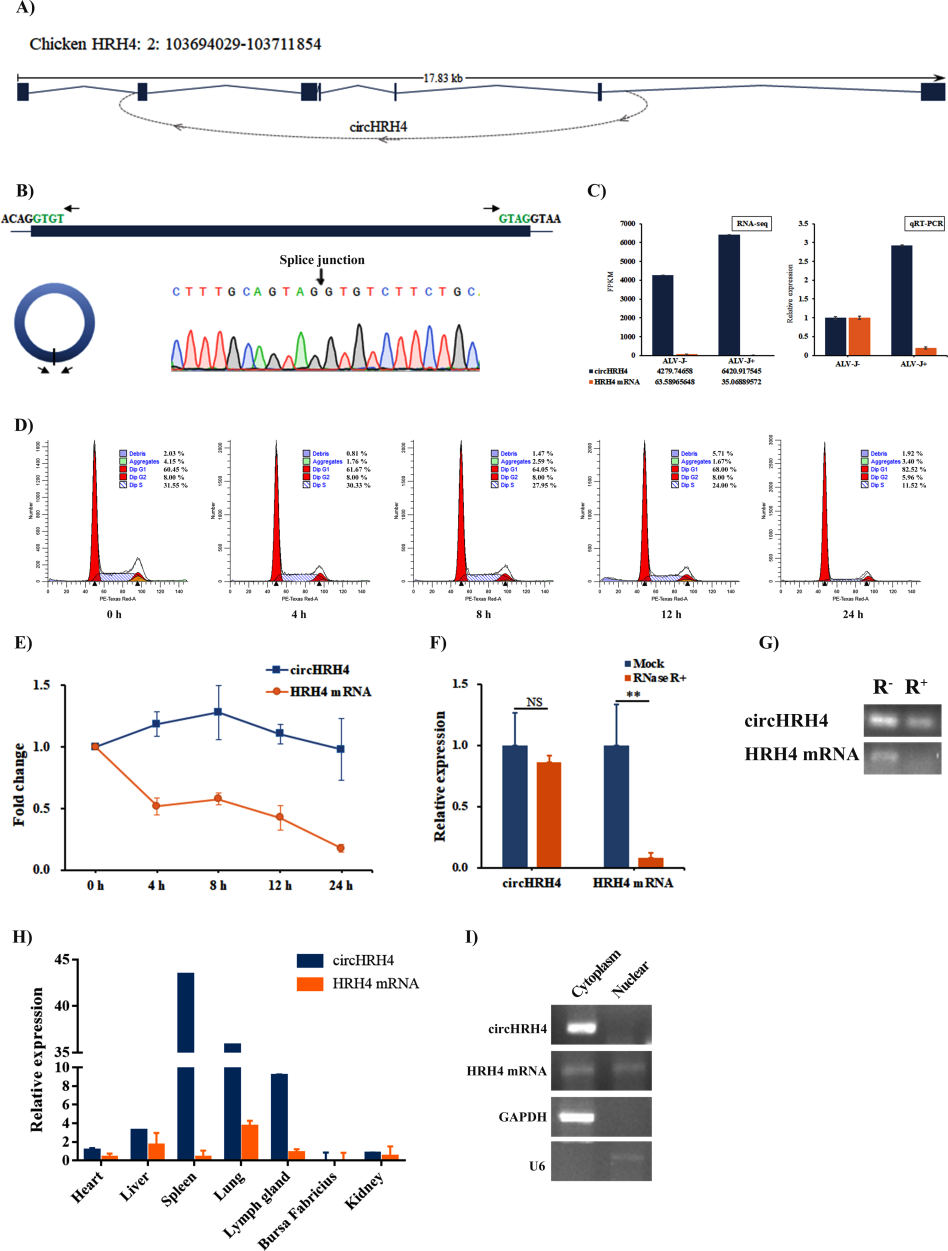
Characterization of circHRH4 RNA in chicken. **(A)** The genomic loci of circHRH4 in *HRH4* gene. **(B)** The expression of HRH4 was validated by qRT-PCR using divergent primers follwed by Sanger sequencing. Arrows represent divergent primers binding to the genome region of circHRH4, and arrow above peak figure represent the splice junction of circHRH4. **(C)** RNA-seq and qRT-PCR results for circHRH4 and *HRH4* mRNA in chicken spleen tissues with or without ALV-J infection. **(D)** Analysis of cell cycle distribution in cells at different time points after Actinomycin D. **(E)** qRT-PCR analysis for the abundance of circRH4 and HRH4 mRNA in DF-1 cells treated with Actinomycin D at different time points. **(F)** qRT-PCR analysis for the abundance of circRH4 and HRH4 mRNA after treated with RNase R exonuclease and the amount of circHRH4 and HRH4 mRNA were normalized to the value measured in the mock treatment. **(G)** Agarose gel electrophoresis for the detection of circHRH4 and HRH4 mRNA with or without RNase R treatment. R^-^: mock treatment, R^+^: RNase R treatment. **(H)** qRT-PCR for circHRH4 and HRH4 mRNA in seven chicken normal tissues. **(I)** qRT-PCR analysis of nuclear and cytoplasmic circHRH4 RNA and HRH4 mRNA in HD11 cells.

We further noted that one of these circRNAs (circRNA_1193, we termed circHRH4), derived from *Hrh4* gene, was particularly abundant and targeted *Mll* and *Aoc3* cooperating with miRNAs in the network. The genomic structure shows that circHRH4 is an exon-intron circRNA, contains four exons and two long complete introns from the HRH4 gene flanked by partial introns on either side (total 6739 bp) (Fig 5A). The product of the expected size was amplified using divergent primers and confirmed by Sanger sequencing (Fig 5B), and the arrow above peak figure represent the splice junction of circHRH4. Consistent with the RNA-seq results, circHRH4 was more abundant in spleen tissue than the linear form (HRH4 mRNA) in chicken as indicated by qRT-PCR analysis (Fig 5C). Tissue expressional profile of circHRH4 and HRH4 mRNA showed that circHRH4 was also significantly more abundant in various tissues (except for bursa Fabricius and kidney tissues) than the linear form (Fig 5H) and circHRH4 is commonly expressed in various tissues and particularly enriched in the spleen and lung. We then investigated the stability and localization of circHRH4 in HD11 cells. Total RNA was harvested at 0, 4, 8, 12 and 24 h post treatment with Actinomycin D, which is an inhibitor of transcription. The cell cycle analysis of cells at different time points showed that Actinomycin D inhibited G1 phase cells from entering S phase and inhibits RNA synthesis (Fig 5D). Analysis of circHRH4 and HRH4 mRNA revealed that circHRH4 was highly stable, with transcript half-life more than 24 h, while HRH4 mRNA with transcript half-life of 4 h (Fig 5E). Further confirmation of circHRH4 existence in chicken was conducted by digesting spleen RNA with RNase R exonuclease. qRT-PCR analysis RNA with or without RNsae R treatment showed that resistence to digestion with RNase R of circHRH4 was high, whereas the associated linear transcript (HRH4 mRNA) degraded significantly (Fig 5F and 5G). qRT-PCR analysis of nuclear and cytoplasmic circHRH4 RNA demonstrated that the circular form of HRH4 localized in the cytoplasm (Fig 5I). Taken all together, our results show that circHRH4 is an abundant and stable circRNA expressed in different tissues and cells in chicken.

## Discussion

ALV-J has caused severe tumor burdens in chickens [10], and it is also a key co-infection factor in avian dieases [45]. Though ALV-J has been eradicated from breeding flocks successfully in the western world, it has become more pervasive throughout China in recent decades [6].

To explore key genes, circRNAs and ceRNA networks involved in ALV-J-induced tumorigenesis, in this study, spleen tissues of six 20-week-old female BSFs with (n = 3) and without (n = 3) spontaneous ALV-J infection were subjected to RNA-Seq and small RNA-Seq analyses. We generated a systematic annotation of the transcriptome of ALV-J-infected and uninfected chicken spleen samples. Transcriptome analysis revealed thousands of circRNAs expressed in ALV-J-infected and uninfected chicken spleen samples. Through differential gene-expression analysis and Venn diagram analysis of mRNAs and host genes of circRNAs, we found eight genes in common: in which four gene are tumor-related, including *Dock4*, *Fmr1*, *Zfhx3*, *Ralb*. These genes have been reported in cancers or tumors in human [34, 37–39].We characterized circRNA catalogs in chickens, compared them with mRNAs and lncRNAs and found that in chickens, circRNAs have relatively shorter transcripts and similar GC content, these characteristics are similar to lncRNAs in chicken in our previous study [46]. We also found that one gene could produce multiple circRNAs in chicken, which is consistent with results in human [21]

Systematic bioinformatics analysis of differentially expressed circRNAs in spleen samples from ALV-J-infected chickens and uninfected chickens revealed several highly significantly enriched GO terms and pathways. These included several immune-associated or tumor-associated terms and pathways, such as pre-B cell differentiation, negative regulation of cysteine-type endopeptidase activity involved in apoptotic process, mTOR signaling pathway, TGF-beta signaling pathway, Toll-like receptor signaling pathway, RIG-I-like receptor signaling patheway, Jak-STAT signaling pathway, Insulin signaling pathway and the ErbB signaling pathway. These results were remarkably similar to previous study on ALV-J-infected HD11s and CEFs [46] and differentially expressed lncRNAs enrichment analysis in ALV-J-infected tissues [32].

The ceRNA network results were based on the differentially expressed of mRNAs and circRNAs associated with ALV-J infection. Of interest, we found 92 circRNA–miRNA–mRNA co-expression links, in which twelve mRNAs, three circRNAs and four miRNAs are involved in tumorigenesis. There are three transcripts of *Mll* gene and one of *Aoc3* gene may be involved in ALV-J-induced tumorigenesis, which have been reported in human by previous studies [42–44].

We characterized one of the abundant circRNAs produced from the *HRH4* gene (we termed it circHRH4). circHRH4 consists of four exons and two long complete introns from the *HRH4* gene and exon skipping was not observed in the *HRH4* gene through RNA-seq data, which indicated that direct back-splicing with intronic RNA pairings may result the formation of circHRH4. Although circRNAs are expressed at low levels generally [47], in this study, RNA-seq and qRT-PCR results showed that circHRH4 was more abundant than the linear form (HRH4 mRNA) in various tissues in chicken, which is also found in other studies [48, 49], the lower levels of associated linear mRNAs may result from circRNA biogenesis competing with pre-mRNA splicing [47, 50]. In addition, the expression patterns of circRNAs are diverse among tissues in chicken, which is same with mammals [51]. Actinomycin D and RNase R exonuclease treatment assays showed that circHRH4 is an abundant and stable circRNA expressed in different tissues and cells in chicken. Further study will focus on experimental identification and characterization of circHRH4 and its associated molecules and their functional evaluation in ALV-J-infected cells and individuals, and validation of the roles of *Dock4*, *Fmr1*, *Zfhx3*, *Ralb*, *Mll* and *Aoc3* in ALV-J-induced tumorigenesis and the relationships between *Mll*, *Aoc3* and their related circRNAs, miRNAs, and the ceRNA networks in the ALV-J-induced tumorigenesis in chicken.

## Supporting information

S1 Table Details of these significantly enriched GO terms of differentially expressed circRNAs between ALV-J-infected and uninfected chickens. (XLS)

S2 Table Details of these significantly enriched pathways of differentially expressed circRNAs between ALV-J-infected and uninfected chickens. (XLS)
